# Ecosystem Resilience and Threshold Response in the Galápagos Coastal Zone

**DOI:** 10.1371/journal.pone.0022376

**Published:** 2011-07-21

**Authors:** Alistair W. R. Seddon, Cynthia A. Froyd, Melanie J. Leng, Glenn A. Milne, Katherine J. Willis

**Affiliations:** 1 Long-Term Ecology Laboratory, Biodiversity Institute at the Oxford Martin School, Department of Zoology, University of Oxford, Oxford, United Kingdom; 2 Department of Geography, Swansea University, Swansea, United Kingdom; 3 NERC Isotope Geosciences Laboratory, British Geological Survey, Keyworth, Nottingham, United Kingdom; 4 Department of Earth Sciences, University of Ottawa, Marion Hall, Ottawa, Ontario, Canada; 5 Department of Biology, University of Bergen, Bergen, Norway; Argonne National Laboratory, United States of America

## Abstract

**Background:**

The Intergovernmental Panel on Climate Change (IPCC) provides a conservative estimate on rates of sea-level rise of 3.8 mm yr^−1^ at the end of the 21^st^ century, which may have a detrimental effect on ecologically important mangrove ecosystems. Understanding factors influencing the long-term resilience of these communities is critical but poorly understood. We investigate ecological resilience in a coastal mangrove community from the Galápagos Islands over the last 2700 years using three research questions: What are the ‘fast and slow’ processes operating in the coastal zone? Is there evidence for a threshold response? How can the past inform us about the resilience of the modern system?

**Methodology/Principal Findings:**

Palaeoecological methods (AMS radiocarbon dating, stable carbon isotopes (δ^13^C)) were used to reconstruct sedimentation rates and ecological change over the past 2,700 years at Diablas lagoon, Isabela, Galápagos. Bulk geochemical analysis was also used to determine local environmental changes, and salinity was reconstructed using a diatom transfer function. Changes in relative sea level (RSL) were estimated using a glacio-isostatic adjustment model. Non-linear behaviour was observed in the Diablas mangrove ecosystem as it responded to increased salinities following exposure to tidal inundations. A negative feedback was observed which enabled the mangrove canopy to accrete vertically, but disturbances may have opened up the canopy and contributed to an erosion of resilience over time. A combination of drier climatic conditions and a slight fall in RSL then resulted in a threshold response, from a mangrove community to a microbial mat.

**Conclusions/Significance:**

Palaeoecological records can provide important information on the nature of non-linear behaviour by identifying thresholds within ecological systems, and in outlining responses to ‘fast’ and ‘slow’ environmental change between alternative stable states. This study highlights the need to incorporate a long-term ecological perspective when designing strategies for maximizing coastal resilience.

## Introduction

Tropical mangrove ecosystems provide essential economic, geomorphological, and ecological ecosystem services by stabilizing eroding coastlines and offering protection from extreme storm surge and tsunami events. They also provide nurseries for economically valuable fishes and crustaceans, and a fuel wood source to local populations [1], [2]. Mangroves are well adapted to the dynamic coastal zone; they can respond rapidly to environmental disturbance and tolerate episodic inundations of salt water and sediment movement. However, mangroves are under threat from anthropogenic land degradation and the combined impacts of climate change. Sea level rise is predicted to increase to 3.8 mm yr^−1^ due to the thermal expansion of water and glacial melting and climate change scenarios also predict an increase in the frequency and magnitude of extreme climatic events [3]. As a result, many mangrove ecosystems may be approaching critical thresholds for healthy functioning and it is necessary to understand how they will respond to the impacts of future environmental change [2], [4].

Mangrove ecosystems consist of a series of vegetation communities dependent on an elevation gradient, comprised of mangroves, saltmarsh, and cyanobacterial microbial mats which are organized in parallel zones along the coast [5], [6]. Because the location of these zones are principally controlled by tidal elevation, any disturbance linked to changes in tidal height or amplitude will result in an adaptation and ecological response [7], [8]. The form of ecological response can vary, and can include: (i) increased sediment deposition and a resultant rise in the relative elevation of mangrove stands in response to rising sea levels; (ii) ecological compositional change; and (iii) horizontal migration upslope and inland [8]. Disturbances can arise from either large external perturbations such as tsunami and storm surge events, or from smaller changes such as variations in salinity due to fluctuating tidal levels [9]. Climate change can also alter local hydrological conditions [7], whilst internal competitive relationships within mangrove communities can enhance or reduce the ability of the ecosystem to resist change [10]. These processes are likely to interact on a hierarchy of temporal and spatial scales, leading to non-linear behaviour that is difficult to model or predict [4], [11].

The ability of an ecosystem to ‘tolerate or adapt to disturbance without collapsing into a different or qualitative state’ [12] is an emergent property known as ecosystem resilience [13], [14]. Resilience theory provides a framework for scientists to interpret the non-linear dynamics of a natural system and understand the mechanisms of abrupt ecosystem change [15]. Resilient systems require large external perturbations to be driven across a threshold into an alternative stable state. Their high resilience is a result of a negative feedback response to a series of ‘fast-small’ environmental processes operating at the local scale; these feedbacks maintain the system in equilibrium and allow it to resist change. Non-resilient systems result from the accumulation of ‘slow-large’ processes, which are influential on larger spatial scales and reduce the resilience over time. In non-resilient systems, thresholds are relatively lower and ‘catastrophic regime shifts’ occur more readily, resulting in new stable equilibria and new landscape conditions [16].

Resilience is a critical concept in contemporary ecology and has been applied at the local, regional, and global scale [15]–[17], providing a useful framework for conceptualizing emergent behaviour and in understanding complex responses to environmental change [14], [18]. At present, the importance of understanding resilience with respect to future environmental changes in the coastal zone has been identified [2], but there is currently a poor understanding of the long-term (>1000 years) links between resilience and ecological change in mangrove ecosystems. Similarly, although non-linear ecological behaviour in mangroves has been proposed [4], there are few studies which have attempted to identify or explain thresholds of ecological response.

In this study we investigate ecological resilience in a mangrove ecosystem from the Galápagos Islands. We use stable carbon isotopes (δ^13^C) and AMS radiocarbon dating to examine two key ecological responses in mangrove systems – community compositional change and increasing accumulation rates – to environmental changes over the past 2,700 years. Past changes in salinity are reconstructed using a diatom transfer function in order to estimate short-term tidal disturbances. Geochemical data is used to examine the long-term environmental changes that occurred at the coastal site. and results are also compared to high-resolution palaeoclimatic data from a nearby crater lake [19]. Global scale geophysical modelling is also employed to predict longer term estimates of relative sea-level (RSL) change as a result of post-glacial isostatic adjustment (GIA). In this study we investigate three key questions: What were the ‘fast and slow’ processes operating in the coastal zone? Is there evidence for a threshold response? How can the past inform us about the resilience of the modern system?

### Study Site

**Figure 1:**
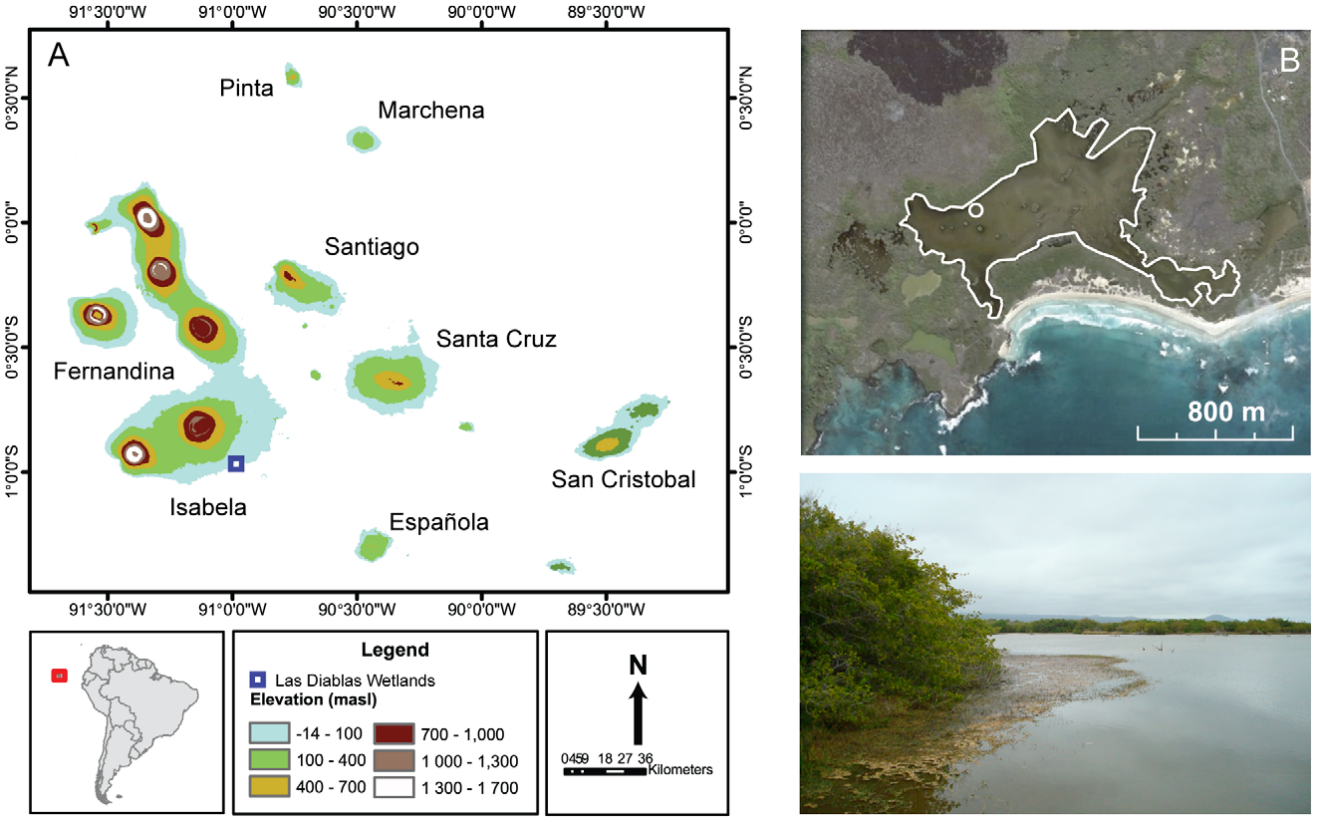
Map satellite image and photograph of the Diablas coastal lagoon. (A) Map of the Galápagos Islands. Diablas lagoon is located in the Diablas wetlands on the south side of Isla Isabela. (B) Satellite image of the Diablas wetlands. The coring location is marked with a circle on both satellite images and the lagoon outline has been traced (*Source: GoogleEarth* © *2010*). (C) View from the far-east side of Diablas lagoon, Isabela, looking NW.

Diablas lagoon is a 79 ha coastal lagoon situated on the south coast of the island of Isabela (0°57′6.51″S, 90°59′9.69″W). The lagoon is at sea level, and is separated from the sea by a 6 m high mangrove boundary and sandy beach, 10–300 m in width (Fig. 1). Three mangrove species, *Conocarpus erecta*, *Rhizophora mangle* and *Laguncularia racemosa,* occur around the lagoon. The maximum water depth is 1.5 m along the northern shore, although most of the lagoon is approximately 30 cm deep. The contemporary lagoon sediment is composed of a pink, gelatinous, microbial mat. Salinity varies between 7–9 g L^−1^, and the pH is 8.4 [20]. Between April and December the Galápagos coastal zone is arid with cooler temperatures due to the cool Humboldt current from the south east. During the wet-season between January and March the southward migration of the ITCZ causes warmer temperatures and intense, daily equatorial showers, whilst El Niño events are an enhancement of the wet season conditions [21]. Due to the negative precipitation/evaporation balance of the Galápagos coastal zone, the low brackish salinities indicate that the lagoon is fed from a freshwater spring with an aperiodic mixing of sea-water.

## Materials and Methods

### Sediment samples

A 4.9 m sediment core was taken in 0.8 m of water in Diablas lagoon, close to the northern fringe of *Rhizophora mangle,* using a Livingstone piston corer [22] (Fig. 1). Samples were wrapped in plastic and foil, transported back to the laboratory, and refrigerated at 4°C to await subsampling. We also collected samples of the possible sources of autochthonous organic material (e.g. mangrove plants, microbial mats) to aid the interpretation of down core variations in carbon isotope composition.

### Core chronology and sediment accumulation rates

Age chronologies for each sediment sequence were developed from eight samples using bulk accelerator mass spectrometry (AMS) radiocarbon dating (Table 1). Calibration and age-depth modelling was undertaken in CLAM [23] using the IntCal04 Southern Hemisphere Curve [24]. CLAM is a freely available package written for the R programming language [25]. ^14^C ages were reported as conventional radiocarbon years BP (AD 1950) and calibrated age ranges were reported as 2σ Weighted Average standard deviations of the calibrated files, rounded to the nearest 5-year interval (Table 1). This calibration assumes no influence from marine carbon due to the reequilibriation of CO_2_ from the atmosphere in the shallow lagoon waters (approx 1 m deep) [26] where, as a result, even submerged aquatic lagoon samples would have acquired the bulk of their organic carbon from atmospheric sources. A smooth spline interpolation method was used for age-depth modelling (1000 iterations). Wet sediment accumulation rates without density corrections were estimated by calculating the slope of the curve at the sampling point for each iteration, and errors were calculated at the 95% confidence intervals at the highest and lowest posterior densities.

**Table 1:** AMS radiocarbon calibration results.

Laboratory Code	Depth (cm)	Lab ID	Modern Carbon (%)	δ^13^C (‰)	^14^C age (yr BP)	2σ Cal. Age (yr BP)
OZI797	56	OXR-1	95.7±0.5	−10.7	350±40	285–485
UBA11498	144	ISD_144	87.7±0.2	−12.1	1051±17	860–970
UBA1499	146	ISD_146	877.2±±0.2	−24.5	1098±17	910–990
OZI798	240–242	OXR-2	83.7±0.6	−24.0	1430±60	1165–1405
UBA11500	311	ISD_311	78.9±0.2	−23.5	1908±18	1705–1865
UBA11501	387	ISD_387	76.9±0.2	−28.6	2114±18	1935–2115
OZI1799	440-443	OXR-3	75.7±0.4	−28.6	2240±18	2070–2380
UBA-14628	483	ISD_483	72.34±0.3	−26.4	2601±28	2470–2790

Bulk sediment accelerator mass spectrometry (AMS) radiocarbon results calibrated using the IntCal04 Southern Hemisphere Curve [24]. ^14^C ages are reported as conventional radiocarbon years BP (AD 1950). Calibrated age ranges are reported as 2σ Weighted Average standard deviations of the calibrated files. These ages are rounded to the nearest 5-year interval. This calibration assumes no influence from marine carbon due to the reequilibriation of CO_2_ from the atmosphere in the shallow lagoon water (max 1 m deep) [26]. As a result even submerged aquatic lagoon samples would have acquired the bulk of their organic carbon from atmospheric sources.

### Carbon isotope ratios

**Figure 2:**
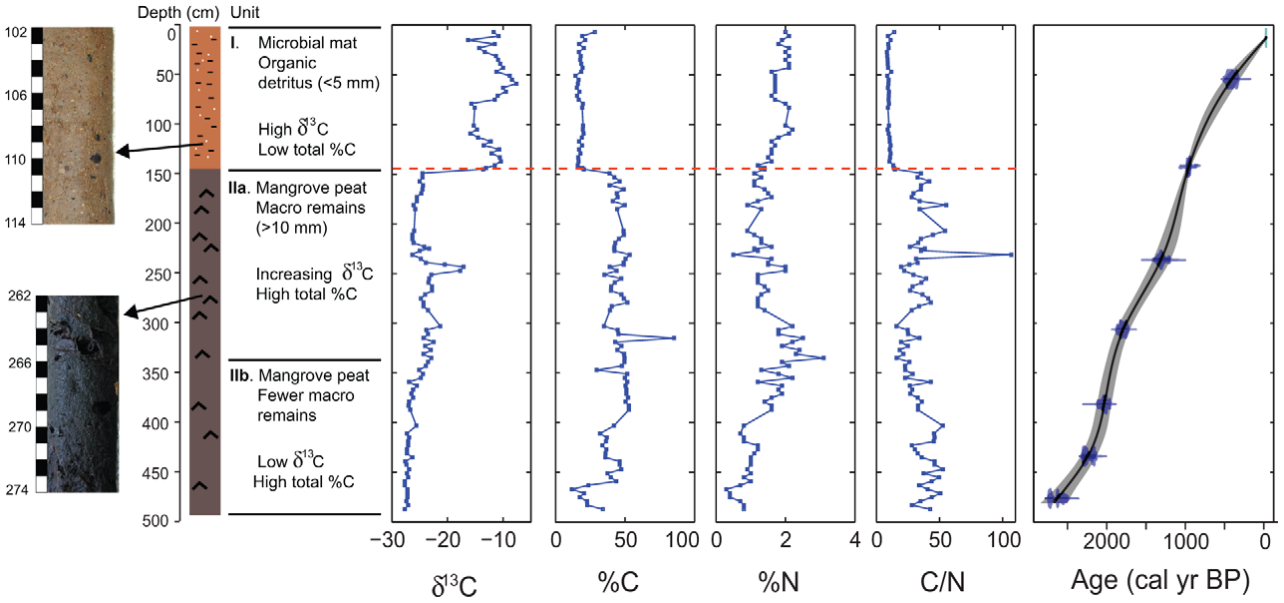
Age-depth, organic and stratigraphic changes observed in the Diablas core.

The carbon isotope signature was used to detect major ecological shifts in autochthonous productivity (Fig. 2). Organic composition (both carbon isotope and C/N ratios) are a function of the sources of organic material of the basin, which can result from either aquatic (fresh or brackish) *in situ* productivity or allogenic material originating from the terrestrial or marine environment [27]. In tropical lagoons, thick organic deposits are likely to accrete as a result of the development of mangrove peats [2] or microbial mats [26]. The plants from these two sources of detrital input have different photosynthetic pathways and therefore different isotopic signatures; in general C_3_ plants, such as mangroves, are isotopically heavier than microbial mats found in alkaline ponds since they preferentially assimilate the lighter isotope ^12^C from the atmosphere.

Carbon isotope composition (δ^13^C) and C/N of the surface and core samples were measured at the NERC Isotope Geoscience Laboratory, British Geological Survey. A sampling resolution of 4 cm was used. Samples were washed in 1 mol HCl to remove carbonates, rinsed with distilled water and sieved under a vacuum using 2 µm filter paper. They were dried, crushed and then weighed into tin capsules. ^13^C/^12^C analyses were performed by combustion in a Costech Elemental Analyser (EA) on-line to a VG TripleTrap and Optima dual-inlet mass spectrometer, with δ^13^C values calculated to the VPDB scale using a within-run laboratory standards calibrated against NBS18, NBS-19 and NBS-22. Replicate analysis of well-mixed samples indicated a precision of ± <0.1‰ (1 SD). C/N ratios can also by measured if required, and these are calibrated against an Acetanilide standard. Replicate analysis of well-mixed samples indicated a precision of ± <0.1.

### Stable isotope mixing model

**Figure 3:**
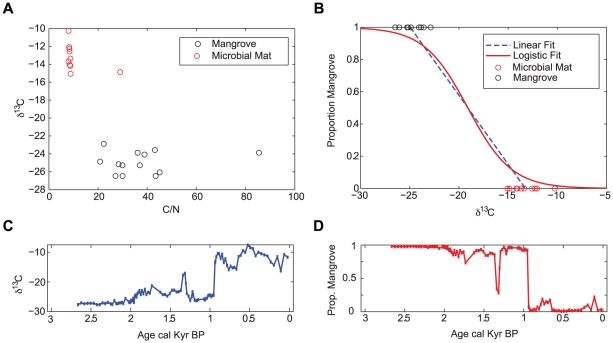
Stable isotope mixing model. (A) C/N and δ^13^C results of surface samples of mangroves and microbial mats in Diablas; (B) Comparison between linear fitting (dashed line) and the logistic curve (full line) fitting methodology used in our model. (C) Stable isotope values and (D) mixing model output for Diablas lagoon.

A stable isotope mixing model [28], [29] was used to determine the proportion of mangroves vs. microbial mat derived organic carbon in the down core samples based on δ^13^C stable isotope measurements (Fig. 3). Surface samples were collected from mangroves and microbial mats growing in the contemporary lagoon environment and measured for their δ^13^C signature. After correcting for the anthropogenic ^13^C Suess effect using mean monthly data (standard curve fitted) from the Mauna Laua observatory, Hawaii. [30] and for decompositional processes by −1.9‰ (the middle value of the range cited by Chumra et al 1987), δ^13^C values for mangroves were determined to range between −26.4 and −22.9‰ and were assigned a proportion value of 1. Microbial mats contained isotopically heavier carbon, with δ^13^C signatures ranging between −16.0 and −10.7‰ and were assigned a proportion value of 0 (Fig. 3b) (mean = −13.2‰).

A logistic curve was fitted to these values using least squares regression (Fig. 3b) (*k* = −0.45, *x^0^* = −19.1, RMSE = 0.038;). Previous studies have used linear mixing models for this purpose [28], [29], but are limited by the fact that core samples with values above (below) the maximum (minimum) mean endmember values result in proportions greater than 1 (less than 0) (Fig. 3b, dashed line). The logistic function overcomes this problem by providing an S- shaped curve which maintains all proportional values between 1 and 0.

In this model, the dominant source of organic material is assumed to be autochthonous. Therefore, the model reflects the accretion of organic material growing in the lagoon at the sampling point. In terms of biological distributions, an index value of 0 indicates an open water lagoon with no mangroves living at the coring site. Conversely, an index value of 1 indicates that the site was completely covered by mangroves above the surface; i.e an expansion of mangroves growing within the lagoon.

### Geochemistry

**Figure 4:**
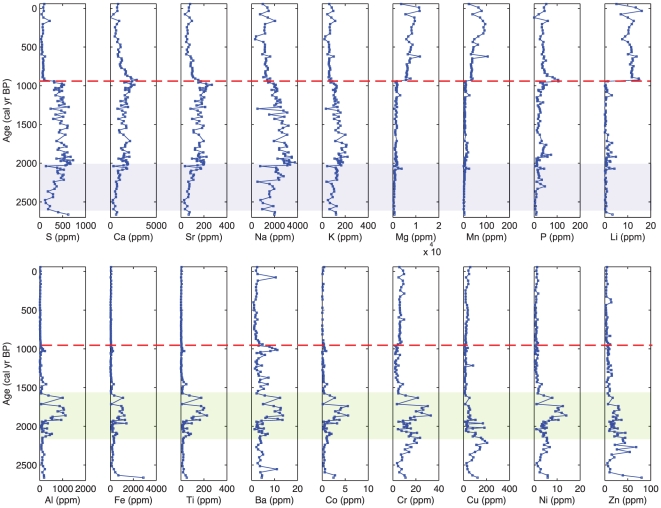
ICP-AES geochemical data from the Diablas lagoon core. Geochemical variations in 18 rare earth elements from the core from the Diablas lagoon. The dashed-red line denotes the major sediment transition identified in figure 2. Blue-shaded area (2700−1990 cal yr BP) indicates the point at which S and associated elements increased, and may indicate increasing tidal influence at the site. Green-shaded area denotes the period (2100−1600 cal yr BP) when geochemical in-wash element indicators (Al, Ti and Fe) were found in higher concentrations in the lagoon and are thought to be indicative of tidal disturbances.

Changes in the abundance of elements in a sediment profile can be used to infer past changes in palaeoecological and palaeoclimatic conditions (Fig. 4) [31]. Traces of geochemical elements in sediments can be used to detect processes occurring both outside of the lagoon catchment (e.g input of tidal influx) and those occurring as a result of *in situ* chemical processes (e.g. chemical changes affecting elements in the sedimentary column after burial) [32]. These techniques have been applied to interpret past environmental changes in a number of coastal systems [33], [34], [35].

Traces of geochemical elemental concentrations were measured at 4 cm intervals on 0.2 g of dry sediment using ICP-AES (Inductively Coupled Plasma-Atomic Emission Spectrometer) after applying a bulk sediment digestion technique [36]. This analysis was undertaken on a Perkin Elmer Optima 3300RL operated by the NERC ICP-AES Facility, Department of Geology, Royal Holloway, University of London.

### Salinity

Salinity changes were estimated using a Weighted Average (WA) diatom transfer function with downweighting for rare species [37], [38] and sample specific errors were estimated by bootstrapping 1,000 samples in RIOJA [39], a freely available package written for in the R programming language [25] (Fig. S1, S2). A total of 40 diatom surface samples from nine Galápagos coastal lagoons were combined with the MOLTEN training set to create a salinity transfer function (Text S1).

One cm^3^ diatom samples were taken from both surface material and from sediment cores. The sediment core was subsampled at a resolution of 4 cm. Samples were prepared using the standard diatom digestion preparation procedures [40], the solution was evaporated onto coverslips before being mounted on slides using Naphrax®. Between 300 and 500 valves of all diatom species present were counted.

### Glacial Isostatic Adjustment Model

**Figure 5:**
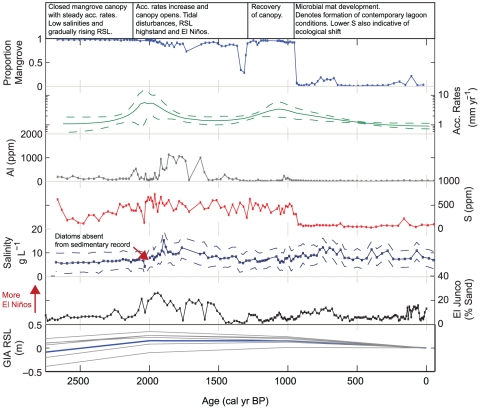
Reconstruction of ecological changes and environmental changes at Diablas lagoon for the past 2,700 years. Note that for the RSL data, seven different modelling parameters are provided (see methods section). The medium parameter model is marked by the thick-blue line. Grain size data is from El Junco crater-lake, San Cristobal (see ref. 19).

Shorelines in the Galápagos have varied on glacial-interglacial timescales, with sea levels approximately 120 m lower during the Last-Glacial Maximum (LGM) due to an increased proportion of the global water budget locked in terrestrial ice caps [41]. Melting of the ice caps results in changes in relative sea level which is not spatially uniform due to GIA [42]. Output from a GIA model was used to determine the past changes in RSL (Fig. 5). The model has two key components: a model of global ice distribution and a model of Earth properties (e.g. density, elasticity and viscosity). The results shown in Fig. 5 are based on a recently developed ice model [43] and a suite of seven Earth models chosen to sample a range of values for three different parameters often inferred in GIA modelling studies: lithospheric thickness (the thickness of an elastic layer at the model Earth surface), viscosity in the upper mantle (the base of lithosphere to seismic velocity jump at 660 km depth) and viscosity in the lower mantle (660 km to core-mantle boundary). The range of values considered for these parameters were, respectively: 71, 96 & 120 km; 0.1, 0.5 & 1×10^21^ Pas; 1, 10 & 50×10^21^ Pas. These ranges accommodate the majority of plausible values for most locations.

### Climate

Climatic conditions in the Galápagos are driven by variability in the El Niño Southern Oscillation (ENSO), which results in a close correlation in weather patterns across different lowland sites (Fig. 5). Data from a high-resolution (∼25 years) palaeoclimate record, based on sediment grain size data as a measure of erosional in-wash from a sedimentary sequence from El Junco crater lake, San Cristobal [19] was plotted against the proxy data obtained in this study.

## Results

### Core stratigraphy

The core is separated into two major stratigraphic sections, an upper section (top-145 cm) of composed of pink, gelatinous sediment which is indicative of a microbial mat (Fig. 2). This top section is homogenous in character, but is mottled with some smaller (approx 1–5 mm) black (organic) detritus and white inorganic particles (<1 mm) which probably are likely to represent gypsum precipitates, commonly observed in shallow water, saline sediments in arid locations [44]. The basal section (145–490 cm) is composed of a dark, organic peat sediment with macroscopic plant remains, roots and shoots, which likely represents a mangrove peat commonly found to accumulate in tropical lagoon locations [45]. This section can be further divided at 323 cm, with fewer large (>10 mm) plant remains in zone 2b at the base. Throughout section 2 there is limited stratigraphic evidence of inorganic material.

### Core chronology

Radiocarbon dating results are provided in Table 1, and indicate that the sedimentary sequence spans the last 2,700 years (Fig. 2). Between 2670–2200, 1650–1200, and 800 cal yr BP to present, sedimentation rates were low and steady (approximately 1–2 mm yr^−1^), but the record is punctuated with periods of higher sedimentation rates, peaking at 2050 (5.7 mm yr^−1^) and 1060 cal yr BP (3.4 mm yr^−1^). No hiatuses are apparent in the sedimentary record indicating continuous deposition of organic matter through time. In general, these sedimentation rates are in line with other late Holocene mangrove sequences, which accrete between 1.5–2 mm yr^−1^ [45].

### Carbon isotope ratios

There is a transition from low (approx – 27‰) to high (approx – 12‰) δ^13^C at 940 cal yr BP (Fig 2a). Between 2670 and 940 cal yr BP the core has a low δ^13^C and a higher proportion of organic carbon, but between 1960–1270 cal yr BP, deviations towards higher δ^13^C occur. The stable isotope mixing model (Fig. 3) indicates four key periods of ecological change at the coring site over the past 2,700 years: (I) a stable mangrove ecosystem between 2670–1960 cal yr BP; (II) slow and steady benthic microbial mat development between 1960–1270 cal yr BP; (III) mangrove ecosystem recovery between 1270–940 cal yr BP; and (IV) an abrupt ecological transition from mangroves to microbial mat (940 cal yr BP), which was dominant for the remainder of the record.

### Geochemistry

The major stratigraphic and isotopic changes observed above are easily identifiable in the geochemical signature of the Diablas core (Fig. 4). Na, S, Ca and Sr began to increase in concentrations from 2550 cal yr BP and remained high until 940 cal yr BP (Fig. 4). The enrichment of S and Ca may be due formation of early diagenetic mineral phases in the form of sulphides and carbonates, which have been donated to the sediment by the sea-water and therefore indicate the increasing influence of the tide [46]. Seawater is also enriched in Sr with respect to freshwater, which may explain the coincidence of these elements at this time [47]. The peak in S at 1990 cal yr BP is important, since it coincides with the higher salinity period in the lagoon inferred from the diatom record (see below). S is in high abundance throughout the mangrove peat section of the core since it commonly attaches itself to organic material under anoxic conditions [48].

Mg, Mn, and Li were found in higher abundances at the top of the sequence and were therefore associated with the microbial mat. The reduction of Mn is the first decompositional process used to gain energy from organic matter in anaerobic sediments [31], and, as a result, its elemental concentrations tend to be lower when conditions are most reducing. Conversely, enhanced Mn profiles are common in oxic sediments [31]. Since the microbial mat sediments donate oxygen to the sediment surface, it is likely that the switch from low to high Mn at 940 cal yr BP reflects change in the redox conditions at the site, which are associated with a switch from anoxic to oxidized sediments following the transition of a mangrove derived sediment to a microbial mat.

Between 2020–1600 cal yr BP, there were peaks of Fe, Al, and Ti in the sedimentary sequence (Fig. 4). Covariation of these three elements is unlikely to have resulted from diagenetic effects; instead, these elements are generally assumed to be minerogenic in origin due to erosional processes in the surrounding basin [49]. As a result, these elements are therefore likely to represent either high-energy, above ground, tidal surges, or erosional in-wash due to resulting from the period of stronger, more frequent El Niño events [19].

### Salinity

The core is generally composed of benthic brackish water diatoms and experienced a transition of species from the base of the record to the present day (Fig. S2). *Fragilaria* cf. *subsalina* (Grunow) Lange-Bertalot and *Pseudostaurosiropsis geocollegarum* (Witkowski & Lange-Bertalot) Morales were dominant at the base of the record, and indicated stable salinities (approx 3–5 g L^−1^) for 800 years (Fig. 5). Diatom concentrations suddenly dropped to zero in three samples between 2020–1990 cal yr BP, and were replaced by *Achnanthes submarina* Hustedt. This was the dominant taxon in the lagoon between 1990–1600 cal yr BP, where there was peak in lagoon salinity at 15.1±4 g L^−1^. A number of marine diatoms (e.g. *Paralia sulcata* (Ehrenberg) Cleve) were also observed in low abundances (<5%, not included in Fig. S2) in the diatom record at this time. Smaller increases in salinity occurred between 800–600 and 400–300 cal yr BP (Fig. 5) and are indicated by the colonization of *Nitzschia palea* (Kützing) W.Smith, followed by *Navicula galapagoensis* Seddon & Witkowski, and *Amphora acutiuscula* Kützing and *A. caroliniana* Giffen. Salinities have varied between 7–11 g L^−1^ for the last 1,100 years.

### Relative sea level

Output from the GIA model indicates that sea level increased steadily at our study location, and reached a small highstand of between 0.1–0.4 m between 2000 and 1000 cal yr BP. The predicted amplitude of the highstand is dependent on the adopted Earth viscosity model. This pattern of RSL change was observed in all but one of the model runs (that with a relatively high lower mantle viscosity of 5×10^22^1Pas), which showed a steadily rising RSL for the past 3,000 years. The predicted change from a RSL rise to fall is due to the reduction in (predominantly Antarctic) ice melt rate during the late Holocene [50]. As the melt rate decreases, the contribution from isostatic processes becomes dominant, which leads to a RSL fall at this locality. The rate of RSL fall is sensitive to the adopted mantle viscosity values. In general, the greater the viscosity, the slower the rate of deformation and the slower the predicted RSL fall, hence providing a possible explanation for the monotonic RSL rise predicted for the Earth model with a relatively high value of lower mantle viscosity.

## Discussion

### Chronological palaeoenvironmental reconstruction

**Figure 6:**
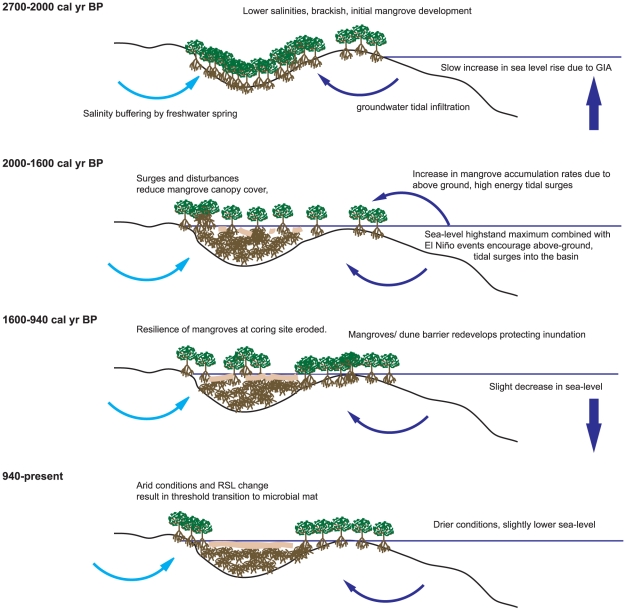
Hypothesised model of environmental changes inferred at Diablas lagoon over the Holocene.

Sedimentation at the coring site in Diablas began at 2,670 cal yr BP and is probably linked to the fact that relative sea levels were approaching their highest since the Last Glacial Maximum [51]. Salinities were stable, between 3–5 g L^−1^ with no marine diatoms present, but there may have been some indirect marine influences through groundwater infiltrating from below. Lithological evidence, combined with the high%C and stable isotope results, suggests that the base of the core was a mangrove peat comprised of refractory roots and leaf litter, which are typically found beneath the canopy of mangrove forests [52], [53], [54]. Thus, at this time, there was a dense mangrove canopy living at the coring surface which accreted at a rate of 1–2 mm yr^−1^ for the first 700 years (Fig. 5). This probably represents the first infilling of a basin as RSL rose (Fig. 6).

2020–1600 cal yr BP was an important period of environmental change at Diablas lagoon. Modelling results indicate that sea levels reached a maximum at around this time (between 0.15–0.4 m above present day levels), whilst a number of shorter term, higher energy events were also observed in the record and are the result of a breaching of a seaward barrier (Fig. 6). For example, no diatoms were found in the sediments between 2020–1990 cal yr BP, which likely indicates a high-energy disturbance event, following which salinities in the lagoon increased to 15 g L^−1^ in only 100 years. Elements such as Al, Fe and Ti were also high between 1960 and 1580 cal yr BP and, since marine diatoms were also found in low abundances in the diatom record at this time, may also be indicative of minerogenic deposition as a result of above ground, higher-energy tidal surges (Fig. 6). Since palaeoclimatic data from the nearby highland El Junco crater lake indicates that this was a period of stronger, more frequent El Niño events [19], ENSO variability may have acted as an additional driving force to these disturbances through low pressure ocean surges (Fig. 5). Alternatively, increases in Al, Fe and Ti may have been driven by ENSO variability directly via precipitation driven erosional inwash.

Despite the increasing exposure to tidal surges, mangroves remained the dominant source of biogenic accumulation at the coring site over this 400 year period. An interesting result is the fact that, at the same time that diatoms were removed from the record through tidal disturbance, and when salinities in the lagoon began to increase dramatically, mangrove accumulation rates had peaked at around 5.7 mm yr^−1^. In this way, the mangroves were able to accrete vertically in line with the tidal inundations, a common response which encourages the stability of the canopy through time (see below).

In addition to this vertical accretion, between 1960–1270 cal yr BP, stable isotope evidence indicates a shift to more variable, heavier isotopes of carbon. Benthic microbial mats are common at the surface of scrub mangrove forests, and it is likely that this represents a reduction or opening of the canopy as following the higher-energy tidal inundations [11]. By 1540 cal yr BP, evidence suggests that the direct effects of the high-energy tidal surges had diminished, and the modelling evidence indicates that RSL was either stable, or had begun to decrease. Sediment accumulation had decreased to 1.4 mm yr^−1^, and salinities to 7.8 g L^−1^ (Fig. 5). Therefore, we propose that, by then, a barrier had begun to develop at the sea-ward side of the coring site which protected the site from tidal inundation and eventually encouraged mangrove recovery (Fig. 6).

At 940 cal yr BP, there was a rapid ecological transition from mangroves to a microbial mat, as evidenced by both stable isotopic, geochemical, and stratigraphic evidence (Fig. 5). This shift indicates an expansion of the microbial mat dominated lagoonal sediments, and resulted in mangroves only being present at the lagoonal fringes (Fig. 6). Diatom evidence from El Junco crater lake indicates cooler sea-surface temperatures at this time, which would have resulted in drier conditions across the coasts of the Galápagos [55], although studies also indicate that this was a period of stronger, more frequent El Niño events [19], [26]. We propose that the combination of drier background climatic conditions and a slightly declining RSL limited the potential for mangrove development within the lagoon basin, and drove the rapid transition to a microbial mat (Fig. 6).

### What were the ‘fast and slow’ processes operating in the coastal zone?

The Diablas wetlands have been highly dynamic for the past 2,700 years experiencing significant environmental and ecological changes linked to GIA, high-energy tidal disturbance events and climatic change. These can be organized into a series of ‘fast’ (<50 years, the lowest average resolution available between three samples in our record) and ‘slow’ (>50 years, i.e. decadal-centennial, e.g. [15]) processes and are described briefly below.

#### Relative Sea Level

Taking the predictions from the GIA model with intermediate parameters, a sea-level highstand of around 0.15 m occurred at 2000 cal yr BP. Since, sea levels were predicted to have changed at an order of magnitude lower than the sedimentation rates observed in our record, the rapid sediment accumulation rates must be associated with other, long-term, geomorphological processes. It is likely that the 4.9 m accumulation resulted from the infilling of the basin as it experienced increasing tidal influence and RSL rise (Fig. 6), whilst the subsequent ‘slow’ fall in sea level (0.15–0.4 mm yr^−1^) following the predicted highstand helped drive the transition from mangrove to microbial mat (Fig. 6).

Alternatively, some of the additional sediment accumulation may also have resulted from tectonic subsidence. This is common in volcanic archipelagos such as the Galápagos, where active islands to the west of the archipelago (Isabela) can experience local-regional scale changes in land elevation through gradual subsidence or sudden landslip events [56]. Here, subsidence of the land may have resulted in a more rapid rise in RSL compared to that predicted by the adopted GIA model (which does not account for tectonic processes). However, this possibility is difficult to test with our data.

#### Tidal Disturbance Events

The system was also punctuated by a series of higher-energy disturbance events associated with above-ground tidal surges (Fig. 5). At around 2000 cal yr BP relative sea levels were at their maximum, disturbances removed diatoms from the record, and lagoon salinities increased to 15 gL^−1^. Geochemical data may then suggest that the lagoon was subjected to similar high-energy events for approximately the next 400 years. These ‘fast’ tidal disturbances were also coincident with a period of increased and more frequent El Niño events, a possible driving force behind the tidal surges at the study site (Fig. 6).

#### Precipitation changes

Palaeoclimatic evidence from El Junco crater lake from the highlands of San Cristobal indicate frequent changes in the strength and magnitude of ENSO over the past 2,700 years, resulting in changes in the precipitation regime on annual-decadal timescales [19]. In the coastal zone, short periods of heavy rainfall (drought) during El Niño (La Niña) events would have reduced (enhanced) lagoon salinities on annual-interannual timescales, but the EEP could also enter El Niño or La Niña phases for hundreds of years. It is likely that the cooler background conditions observed in the EEP between 1000–650 cal yr BP [55] caused increased aridity in the mangrove environment. The accumulation of these drier conditions over time would have increased the aridity stress on the mangroves in the basin, which combined with the slightly decreasing sea level, helped drive the transition from mangrove to microbial mat.

### Is there evidence for a threshold response?

**Figure 7:**
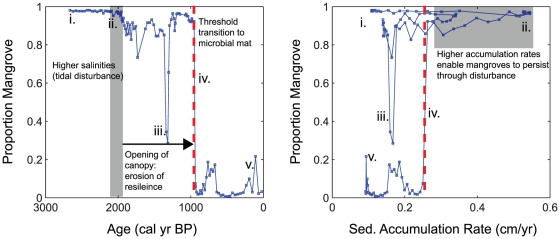
Threshold ecological response at Diablas lagoon. A) Stable isotope mixing model results indicating the main ecological changes at the Diablas lagoon. B) Phase plot to indicate the presence of alternative stable states (mangroves and microbial mat) in the lagoon system. The point at which the threshold between the two states was crossed is marked by a red dashed line. Detailed chronological summary is as follows. (i) 2770 cal yr BP- resilient mangrove stand at the coring site; (ii) 2000 cal yr BP- increase in sediment accumulation rates denotes feedback response of resilient mangroves in response to environmental disturbances; (iii) 1400 cal yr BP- opening of the mangrove canopy due to preceding tidal disturbance events, eroding resilience over time; (iv) (940 cal yr BP) Drier background climatic conditions and slightly decreasing RSL combine, resulting in threshold transition to microbial mat; (v) present day; contemporary lagoon conditions with cyanobacterial mat at the sediment surface.

Non-linear responses to environmental change occur as a result of negative feedback processes which maintain a system in an equilibrium state. A key negative feedback identified in mangrove ecosystems is the accumulation of organic material in response to a tidal influx [11]. In this way, mangrove communities can accrete vertically in line with tidal inundation linked to rising sea levels or infilling of the lagoon basin. Here, we observed a faster rate of sediment accumulation occurring during a period of disturbances during a small sea level highstand, suggesting that the mechanism of vertical accretion in response to tidal inundation was occurring (Fig. 7). This process would have ensured the general stability of the mangrove canopy over time, maintaining mangroves as the dominant system at the coring site.

According to resilience theory, the accumulation of slow processes can result in an erosion of resilience over time, making a system more susceptible to smaller perturbations and environmental changes. We propose that the historical period of disturbances occurring after 2000 cal yr BP, which had the effect of the opening up the mangrove canopy, caused an erosion of resilience at our study site (Fig. 7). A facilitation effect has been observed in experimental studies on mangroves [10], in which a higher number of mangrove species per unit area encouraged long-term persistence. In an open canopy, the facilitation effect of mangroves would have been reduced [10]. This decrease in resilience would have made it more susceptible to smaller scale environmental perturbations.

Indeed, the transition to a microbial mat in Diablas occurred at a time of drier climatic conditions in the EEP [57], and was also coincident with an apparent decrease in RSL. Thus, the combination of a reduced canopy cover, combined with drier conditions and a slightly decreasing sea level, limited mangrove development in the basin. As a result, a microbial mat replaced mangroves at the coring site, and forced a threshold transition into an alternative stable state (Fig. 7).

### How can the past inform us about the resilience of the modern system?

These findings have important consequences in our understanding of the ecological response of mangrove ecosystems to future environmental change. Future predictions on the rates RSL rise linked to climate change are uncertain, but the central estimate of the rate of rise at the end of the 21^st^ century is 3.8 mm yr^−1^ [3]. In mangrove ecosystems which appear to experience low inputs of mineral sediment, organic matter accumulation is key in maintaining their persistence and stability. Studies in Belize and Florida showed that the relative contribution of organic solids to surface accretion was one to three times that of inorganic solids and also indicated biological controls on vertical accretion [58]. Therefore, through the vertical processes described above, mangrove ecological communities were able to accrete at rates comparable to locations with high mineral sedimentation in order to cope with a sea-level rise. At the Diablas wetlands, our data indicates that vertical accretion was a major response to the increased incidence of tidal disturbances, occurring at rates of up to 5.7 mm yr^−1^.

However, in our study the resilience of mangroves was eroded by a series of historical processes and, as a result, a combination of arid conditions and slow RSL change eventually resulted in a threshold transition to an alternative stable state. Thus, our study implies that in this system, understanding the patterns of past historical processes are essential for determining baselines and for investigating ecological responses. The study highlights the need to incorporate a long-term ecological perspective when designing strategies for maximizing coastal resilience.

### Conclusions

Palaeoecology is increasingly being applied as a tool to identify long-term baselines and thresholds in ecosystems at the local-regional scale [59]. In this study, we used palaeoecology to show that tidal inundation, and disturbance can be tolerated by Galápagos coastal communities through complex feedback responses linked to vertical accretion. Sedimentation rates of up to 5.7 mm yr^−1^ were observed in the Diablas ecosystem, and planning procedures which aim to encourage mangrove resilience are essential to ensure the long term provisions of ecosystem services from mangroves in the tropical coastal zone. Paleoecological records can provide important information on the nature of non-linear behaviour, by identifying baselines and thresholds within ecological systems, and in outlining key ecological responses to ‘fast’ and ‘slow’ environmental change between alternative stable states.

## Supporting Information

Figure S1 **Weighted average (WA) diatom transfer function.** Plots to indicate the predictive power and residuals from the WA diatom salinity transfer function used in this study.
(EPS)

Figure S2 **Diatom stratigraphy and salinity transfer function results.** Diatom stratigraphy diagram of key species from both sites and diatom salinity transfer function results. Note that Diablas lagoon was mainly composed of brackish species. As a result, salinity was never greater than approximately 15 g L^−1^.
(EPS)

Text S1 **Weighted average (WA) diatom transfer function.** Description and evaluation of the diatom salinity transfer function used in this study.
(DOC)
