# Supplementary material for: Ecosystem Resilience and Threshold Response in the Galápagos Coastal Zone
Source: PLoS One. 2011 Jul 21;6(7):e22376. doi: 10.1371/journal.pone.0022376 (PMC3141046; doi:10.1371/journal.pone.0022376)
Supplement: Text S1 — Weighted average (WA) diatom transfer function. Description and evaluation of the diatom salinity transfer function used in this study. (DOC) [file pone.0022376.s003.doc]

**Supplementary Information**

**Text S1: Weighted average (WA) transfer function**

**Methods**

RIOJA, a package written for the analysis of data sets from stratigraphic sequences in R [1], was used to calculate changes in salinity using a Weighted Average (WA) diatom transfer function with downweighting for rare species and sample specific errors, estimated by bootstrapping 1,000 samples [2,3]. One cm^3^ samples were taken from both surface material and from sediment cores. The sediment core from the Diablas wetlands was subsampled at a down-core resolution of 4. Samples were prepared using standard diatom digestion preparation procedures [4], and the solution was evaporated onto coverslips before being mounted on slides using Naphrax®. Between 300 and 500 valves of all diatom species present were counted. Rare taxa (< 5%) were excluded from the salinity reconstruction analysis and the species proportions were recalculated accordingly.

Surface sediment samples were taken from a range of benthic microhabitats from eight coastal lagoon sites in Galápagos (40 samples in total). This data was combined with 329 samples from the MOLTEN data set from coastal locations around the Baltic Sea [3] for the development of the transfer function. Overall, there was a good fit between measured salinity values and actual salinity (*r^2^* = 0.89, RMSE = 3.1) (Fig. S2). The results indicate that the transfer function has a higher statistical power at lower salinities between 0 and 20 g L^-1^, but that salinities greater than 30 g L^-1^ may be overestimated using this model. Since the reconstructed salinities were never higher than approximately 15 g L^-1^, we are confident that changes in salinity identified in the record

**Evaluation**

Since a number of species were not observed in the combined modern surface sample data set the salinity reconstructions at some points in the sediment cores should be viewed with caution. In a study in which data was artificially simulated [5], reconstructions in which only <5% of fossil taxa are not found in the modern dataset are likely to be very reliable; 5-10% were found to produce reliable results, whilst 10-25% may be reliable, and >25% is likely to be unreliable. Therefore, we calculated the total percentage of taxa in the fossil sample but not in the modern calibration dataset [6].

Using this classification scheme, salinity reconstructions for approximately the last 1,000 years were deemed very reliable, but there salinity reconstructions between 1000-1200 cal yr BP may suffer from poor overlap between the transfer function and core species. In particular, the species *Nitzschia nana* Grunow (maximum abundance = 47.9%), and *Navicula atwateri* Seddon & Witkowski (maximum abundance = 51.7 %) were found occurring in higher abundances in samples at this time, but are not represented in the surface sample training set. Between 1200 and 2000 cal yr BP, in general these values do not fall below 85%, and between 2000 and 2700 cal yr BP, the percentage overlap values are generally found between 80-90%. In summary, when combined with other multi-proxy evidence in this study we believe the general trends reconstructed are representative of the major salinity shifts occurring in the lagoon over the past 2,700 years.

**References**
